# Assessing Ageist Attitudes: Psychometric Properties of the Fraboni Scale of Ageism in a Population-Based Sample

**DOI:** 10.3390/geriatrics11010002

**Published:** 2025-12-24

**Authors:** Jiri Remr

**Affiliations:** INESAN (Institute for Evaluations and Social Analyses), Sokolovská 351/25, 18600 Prague, Czech Republic; jiri.remr@inesan.eu

**Keywords:** ageism, stereotypes, antilocution, avoidance, discrimination, psychometrics, confirmatory factor analysis

## Abstract

**Background/Objectives:** Ageism is a pervasive form of prejudice that undermines health, social participation, and intergenerational solidarity, yet validated research tools for measuring ageism are lacking in many countries. The Fraboni Scale of Ageism (FSA) is one of the widely used instruments, but its psychometric properties have not previously been examined in the Czech context. This study aimed to translate the 29-item FSA, evaluate its reliability and validity, and describe ageism across generations. **Methods:** A quantitative cross-sectional survey based on face-to-face interviews was conducted in March 2024 among the Czech population aged 15–74 years (n = 1096). Data analysis included descriptive statistics, internal consistency indices (Cronbach’s α, McDonald’s ω, Composite Reliability, Average Variance Extracted), exploratory factor analysis (EFA) on a random half-sample, and confirmatory factor analysis (CFA) on the second half. Construct validity was also examined. **Results:** The Czech FSA showed very good distributional characteristics with no floor or ceiling effects and excellent internal consistency (α = 0.949; subscales α = 0.848–0.898). EFA replicated the original three-factor structure (Antilocution, Avoidance, and Discrimination) explaining 57.6% of variance. CFA supported this structure with good-to-excellent model fit. FSA scores increased systematically from Baby Boomers to Generation Z, indicating higher ageism among younger cohorts. Higher fear of old age, lower education, an earlier subjective boundary of old age, and absence of an older co-resident were associated with higher ageism scores. **Conclusions:** The Czech version of the FSA is a reliable and valid instrument for assessing ageist attitudes in the Czech population. Its robust psychometric properties and sensitivity to theoretically relevant correlates support its use for monitoring ageism, evaluating interventions, and enabling cross-national comparisons in aging research and policy.

## 1. Introduction

Ageism is a significant and globally widespread social construct used to understand attitudes toward older people and to clarify how they are perceived and treated in society [1,2,3,4]. According to Butler [5], who originally defined the concept of ageism, it is “the process of systematically stereotyping and discriminating against people because of their age.” In a conceptual synthesis, Iversen et al. [6] analyzed multiple definitions and proposed to conceptualize ageism as “discrimination or negative or positive prejudices against older people on the basis of their age or because they are perceived as old.”

The above definitions imply that attitudes toward older people can be both positive and negative, yet negative stereotypes are prevalent across many cultures [1,4]. Ageism thus encompasses cognitive (stereotypes), affective (prejudices), and behavioral (discrimination) components of negative attitudes toward other people based on age. Although ageism can affect all age groups, it is most often directed at older adults, who may be reduced to categories such as frail, unadaptable, or incompetent [7,8,9]. Many researchers have turned their attention to age-based stereotypes that represent generalized and often mistaken perceptions of older adults that are considered as social facts even without empirical evidence [10,11,12,13]. Discrimination, as a behavioral manifestation of such stereotypes, frequently limits opportunities for stigmatized groups [14]. Globally, the World Values Survey reported that 60% of respondents across 56 countries believe older people do not receive the respect they deserve [15].

The impacts of ageism are far-reaching. They extend to the labor market, the quality of health and social care, intergenerational relationships, and older adults’ subjective well-being [16]. Available evidence links ageism to poorer mental and physical health and even reduced longevity, as well as heightened social isolation and neglect [1,17,18,19]. In clinical and social care settings, ageist attitudes can contribute to inadequate treatment [20,21], while in workplaces they may encourage early exit from employment or hinder re-employment [22].

As scholarly interest in ageism has grown, measurement tools have proliferated. The first generation of scales focused primarily on the cognitive component, i.e., attitudes and opinions about older people [23,24]. In addition to this Attitudes Toward Old People Scale, other widely used instruments include, among others, the Aging Semantic Differential [25], the Stereotype Scale [26], or Tipton’s Comprehensive Ageism Scale [27]. A systematic review [28] identified 11 instruments covering different approaches to ageism as a construct, and noted substantial variation in the emphasis placed on cognitive, affective, and behavioral components. Among these instruments, the Fraboni Scale of Ageism (FSA) [29] stands out as a comprehensive tool that captures attitudinal as well as behavioral aspects of ageism. Grounded in Allport’s [30] conceptualization of prejudice, the FSA comprises three dimensions:

1. Antilocution, defined as antagonism toward older people based on stereotypes. This factor reflects verbal and attitudinal manifestations of ageism rooted in negative stereotypes operationalized by items like, e.g., “Many old people just live in the past”; “Old people complain more than other people do” or “Many old people are not interested in making new friends preferring instead the circle of friends they have had for years”.
2. Avoidance, capturing the tendency to avoid social contact with older people. This factor represent the behavioral component of ageism associated with a preference to avoid interaction. Typical statements include, e.g., “Old people should find friends their own age” or “I personally would not want to spend much time with an old person”.
3. Discrimination, covering support for unequal treatment of older people or the restriction of older people’s rights. Typical statements include, e.g., “Most old people should not be trusted to take care of infants” or “It is best that old people live where they will not bother anyone”.

The FSA has been repeatedly adapted and tested across different cultural contexts [31,32,33]. Most studies support its three-factor structure, although certain items have proven context-sensitive and were removed or revised in some adaptations (see, e.g., [32,33,34]). The FSA has been validated not only in Western countries [29,35], but also in Israel [31], Korea [32], Turkey [36], and China [33]. Despite this international uptake, the psychometric properties of the FSA have not yet been verified in the Czech population.

The aim of this study was to validate the Czech version of the FSA, assess its reliability, dimensionality, and psychometric properties, and employ it to describe the level of ageism among the Czech population. In this respect, particular attention was paid to intergenerational comparisons, using the FSA to compare the level of ageism across age groups (generations). Research questions and hypotheses were as follows:

1. Do attitudes in the Czech population conform to the original three-dimensional model (antilocution, avoidance, discrimination)?
2. Does the Czech version of the FSA achieve psychometric characteristics that are acceptable for use in this population?
3. Is the Czech population homogeneous in its level of ageism, or do age groups differ?

## 2. Materials and Methods

### 2.1. Participants and Procedures

This study was based on a quantitative cross-sectional design to obtain representative data on attitudes toward old age and ageism in the Czech population, measured with the FSA. Given the focus on intergenerational differences, the sample deliberately included adolescents aged 15–17 and respondents aged 65–74. In line with current approaches to generational research (e.g., [37,38,39]), the cohorts were defined as follows:

- Generation Z: born 1997–2012
- Generation Y: born 1981–1996
- Generation X: born 1965–1980
- Baby Boomers: born 1946–1964

To maximize representativeness, a probability design was implemented using a comprehensive list of dwellings as the sampling frame. Selection reflected the regional distribution and the size structure of settlements in Czechia. Each interviewer received 5–15 addresses and identified respondents within the given dwelling using Kish’s table [40], ensuring random selection of individuals. Beyond standard survey eligibility, no specific inclusion or exclusion criteria were applied. To reduce potential contamination and response conditioning, individuals who reported participating in another survey study within the previous six months were not recruited.

Fieldwork took place in March 2024 and had a form of face-to-face interviews. In total, 2032 people were approached and 1133 agreed to participate, yielding a response rate of 55.8%, which is typical for this mode of data collection. Informed consent was obtained from all respondents prior to the interview; for participants aged 15–17, at least one legal guardian also provided consent.

All data were anonymized in accordance with current legislation and ethical principles of social science research. The respondent contact process, voluntary participation, interview protocol, and all fieldwork procedures adhered to the principles specified in the Helsinki Declaration [41]. Appropriate measures were taken to ensure that respondents could not be identified retrospectively, either directly or indirectly. During data processing, questionnaires underwent multi-stage quality control. Thirty-seven cases were excluded due to substantial inconsistencies. The final dataset thus contains 1096 valid cases. Internal quality assurance included checkbacks for 35% of completed interviews to verify methodological and ethical compliance during fieldwork.

### 2.2. Description of the Sample

The final sample comprised 1096 respondents representing the general population of Czechia. As shown in Table 1, the gender distribution was balanced (50.1% were males and 49.9% were females), closely mirroring the theoretical population and supporting the generalizability of findings. Generation Y constitutes the largest cohort (29.8%), followed by Generation X (29.6%), Baby Boomers (20.8%), and Generation Z (19.8%). This structure provides sufficient coverage across cohorts for intergenerational comparisons while acknowledging unequal cohort sizes. With respect to settlement size, the sample covered all settlement types and matched the distribution of theoretical population. Most respondents lived in smaller towns with 1000–4999 inhabitants (22.7%) and large cities with 100,000+ inhabitants (22.2%), followed by medium-sized towns with 20,000–99,999 inhabitants (19.6%), smaller towns 5000–19,999 (18.5%), and rural areas with less than 1000 residents (17.0%). This distribution allows for contrasts between urban and rural contexts.

### 2.3. Translation of the Instrument

The research instrument comprised the Czech version of the FSA and a set of additional items capturing relevant attitudes, declared behaviors, and respondents’ sociodemographic and socioeconomic characteristics. This design enabled assessment of the FSA’s psychometric properties and analysis of associations between ageist attitudes and broader aspects of social life.

To ensure linguistic and cultural comparability of the Czech version of the Fraboni Scale of Ageism (FSA), we implemented a multi-stage translation and adaptation process consistent with established guidelines [42,43]. In the first phase, two independent forward translations of the original English scale were prepared by bilingual translators with excellent proficiency in both languages. One translator had expertise in psychology and the social sciences, while the other approached the task without prior domain specialization, thereby balancing disciplinary insight with a general linguistic perspective. The two versions were then reconciled by an expert panel, which reached consensus on the final wording of each item. This stage resolved terminological differences, stylistic issues, and subtle linguistic nuances that might influence item comprehension. Next, an independent translator, blinded to the original conducted a back-translation from Czech to English. The back-translation was compared with the original instrument and evaluated for semantic, idiomatic, and conceptual equivalence. Minor textual adjustments were introduced where needed to secure functional equivalence between language versions.

Pilot testing was conducted with 26 respondents spanning varied ages and educational backgrounds. Cognitive interviewing techniques were used to probe respondents’ interpretations, identify ambiguities, and document thought processes for each item. This procedure revealed latent differences in interpretation and supported evaluation of clarity and cultural appropriateness. Based on the pilot, several items were partially reformulated, with careful attention to preserving the original meaning. Adjustments were made only where comprehension was clearly compromised or where cultural context risked misunderstanding; the overarching goal was to maintain content validity and conceptual integrity (e.g., [44]). The finalized Czech FSA was then deployed in the main data collection.

### 2.4. Measures

#### 2.4.1. Fraboni Scale of Ageism

The FSA [29] is a multidimensional instrument comprising 29 statements that assess ageism by capturing both cognitive and affective components of attitudes toward older people. The original validation reported Cronbach’s alpha coefficients for three subscales: antilocution (α = 0.76), avoidance (α = 0.77), and discrimination (α = 0.65), with an overall reliability of α = 0.86.

In this study, respondents answered FSA items on a four-point Likert scale (1 = strongly disagree, 4 = strongly agree). The absence of a neutral midpoint encouraged respondents to take a position. Six items (8, 14, and 21–24) were positively worded and were reverse-coded when computing scores. Total scores range from 29 to 116, with higher scores indicating higher levels of ageism.

Unlike some prior adaptations that shortened the FSA (e.g., [31,33,36]) we retained all 29 items to evaluate the instrument’s psychometric properties in its original form. Item reduction was typically justified by low item–total correlations or cultural inappropriateness of specific statements [31,33]. Here, the full version was essential for a comprehensive test of the factor structure and for assessment of applicability of dimensions in the Czech context.

#### 2.4.2. Auxiliary Variables

To deepen validation and interpretation of FSA results, three auxiliary variables that enable thorough testing of the Czech FSA’s construct and convergent validity were included. Demonstrating systematic relationships between FSA scores and subjective definitions of old age, contact experiences, and emotional responses to aging supports the claim that the instrument validly captures the multidimensional nature of ageism in the Czech environment.

Fear of old age indicates respondents’ anxieties related to aging, and their own aging [45,46]. It operationalizes an affective component closely linked to stereotypes about old age. Higher levels of this fear should be associated with higher FSA scores, particularly on antilocution and discrimination. A positive association would support convergent validity; if the FSA measures ageism, it should correlate with this emotionally charged stance toward seniors.

Presence of an older adult in the household is a dichotomous variable (yes/no) that reflects whether respondents share a household with someone they subjectively perceive as “old”. Its relevance derives from the contact hypothesis [30], which posits that sustained, meaningful contact with stigmatized groups reduces prejudice. Accordingly, respondents living with someone they consider old are expected to report lower FSA scores, especially on avoidance. The reason is that daily interactions can foster empathy and understanding, offering an empirical basis for validity anchored in declared social contact [47,48,49].

Perceived age of older people captures the subjective threshold at which a person is regarded as “old”. As noted by Ayalon et al. [28]; Marques et al. [50], or Turner [51], understandings of elder are socially conditioned and vary with cultural context and personal experience. Including this variable reflects the need to take into consideration individual constructions of old age. For construct validity, respondents with lower subjective thresholds (e.g., 50–60 years) are expected to exhibit higher FSA scores, whereas those who place the threshold higher (e.g., 75 or even higher) should score lower. To enable intergroup comparisons, the answers were categorized to 50–65 years; 66–75 years; 76–80 years; 80+ years. Given its link to stereotypical boundary setting, this variable is particularly informative for the Antilocution dimension.

### 2.5. Data Analysis

Analyses were conducted in IBM SPSS Statistics, Version 26 (IBM Corp., Armonk, NY, USA) and AMOS 24. Descriptive statistics summarized sample characteristics and variable distributions, reporting means (M), standard deviations (SD), skewness, and kurtosis. Score distributions for potential floor and ceiling effects, i.e., concentrations of observations at scale extremes that may indicate limited sensitivity within those ranges [52] were examined.

Bivariate associations were estimated using Pearson’s correlation coefficient for continuous variables and Kendall’s tau-b for ordinal and dichotomous variables. Group differences were tested with independent-samples *t*-tests (two groups) and one-way ANOVA (three or more groups). For all tests, effect sizes (e.g., Cohen’s d, η^2^) alongside 95% confidence intervals are reported. Given the non-normality and ordinal nature of some variables, group differences were tested using non-parametric Mann–Whitney U tests (for two groups) and Kruskal–Wallis tests (for three or more groups), alongside parametric *t*-tests and ANOVA where distributional assumptions were reasonably met.

A rank-based coefficient (Kendall’s tau-b) was appropriate in this study because the FSA items are ordinal Likert-type responses with non-normal distributions. Tau-b explicitly adjusts for ties in both variables, yielding an association estimate that does not assume interval spacing or linearity [53]. This makes it more faithful to the monotonic relationships expected among attitudinal items and less sensitive to outliers than Pearson’s r. Compared with Spearman’s rho, Kendall’s tau-b has a clear probabilistic interpretation (the difference between the probabilities of concordant and discordant pairs) and tends to provide a more conservative, small-sample-efficient measure under ordinal data. Using tau-b therefore aligns the analysis with the measurement level and distributional properties of the FSA indicators, supporting valid inferences about inter-item associations.

Internal consistency was evaluated with a combination of four indices. In addition to Cronbach’s α, which assumes tau-equivalence and uncorrelated errors, we also calculated McDonald’s ω (omega), a more robust reliability estimate when item loadings differ [54]. We further computed Average Variance Extracted (AVE) and Composite Reliability (CR) to assess convergent validity of latent constructs; higher AVE indicates that a factor explains a substantial share of its indicators’ variance [55].

The factor structure was examined in two stages combining exploratory and confirmatory strategies. The full dataset was randomly split into two subsamples (n_1_ = 548, n_2_ = 548) when exploratory factor analysis (EFA) was assessed on the first subsample and confirmatory factor analysis (CFA) was performed on the second one. However, four cases in the first subsample were additionally removed due to random occurrence of missing data. This cross-validation approach is common in scale validation studies (see, e.g., [33,56]).

For the EFA, we used principal component analysis (PCA) with varimax rotation, assuming orthogonal factors [57]. The number of components was determined by Kaiser’s criterion (eigenvalue > 1) and visual inspection of the scree plot [58]. The emerging structure was evaluated based on item loadings; threshold ≥ 0.40, a conventional cutoff (see, e.g., [57]) and, where applicable, inter-factor correlations.

CFA was conducted in AMOS 24 using maximum likelihood estimation. Model adequacy was assessed using a combination of absolute, incremental, and parsimony fit indices. Absolute fit was assessed with χ^2^ and degrees of freedom (df), noting χ^2^’s sensitivity to sample size. Incremental fit was evaluated with the Comparative Fit Index (CFI) and Tucker–Lewis Index (TLI); parsimony was indexed by the Root Mean Square Error of Approximation (RMSEA). Consistent with relevant guidelines, values of CFI ≥ 0.90, TLI ≥ 0.90, and RMSEA ≤ 0.08 were taken as indicative of acceptable fit [59].

## 3. Results

### 3.1. Univariate Statistics

Table 2 shows that the item means are grouped around the mid-scale with SDs mostly 0.74–0.90, indicating adequate dispersion and no evidence of floor/ceiling effects. Skewness is generally small and positive for many items, implying respondents tend to disagree modestly with overtly negative statements; kurtosis values are negative, suggesting broad, light-tailed distributions rather than highly peaked ones. The top-mean items indicate stereotypes and antilocution (see items 1, 5, 28), whereas overt personal avoidance (item 6) is least endorsed. The set of items shows usable variability for discrimination across respondents.

The reported Item–Total Correlations (ITC) were computed against the total FSA score for all 29 items taken together (with appropriate reverse-coding). Accordingly, ITC values reflect how strongly each item aligns with overall ageism, not with any single subscale in isolation. ITC are generally strong, supporting internal coherence. The items implying antilocution and avoidance drive much of the reliable variance. A few items (especially items 16 and 2) underperform, which can invoke safety or accommodation rationales rather than pure prejudice.

Positive skew on avoidance items (e.g., items 6 and 7) indicates that more respondents tend to disagree with these statements, which is consistent with social desirability around interpersonal fairness. Several stereotype items show near-symmetric or negative skew (e.g., items 2 and 19), suggesting greater comfort endorsing structural separation than admitting personal avoidance.

Table 2 portrays an attitude profile where negative stereotypes are relatively normalized, while overt avoidance is less openly admitted. The core indicator of ageism is anchored in stereotype endorsement (antilocution) rather than frank avoidance, mirroring the factor results where antilocution is dominant. Most items integrate well with the total scale.

### 3.2. Exploratory Factor Analysis (Dimensionality of the Scale)

Preliminary diagnostics supported the suitability of the data for factor analysis. The Kaiser–Meyer–Olkin (KMO) index was 0.962, which indicates excellent sampling adequacy [60,61]. This value suggests sufficiently strong and coherent inter-item correlations for factor extraction. Bartlett’s test of sphericity was significant (χ^2^ = 9278.211; df = 406; *p* < 0.001), confirming that the correlation matrix is not an identity matrix.

Using principal components with varimax rotation, three factors were extracted, jointly explaining 57.6% of the total variance. For comparison, Fraboni et al. (1990) [29] reported 52.6% explained variance in the original validation. Contributions of the factors to total variance were as follows (original study values in parentheses): factor 1 (Antilocution) = 41.8% (23.3%); factor 2 (Avoidance) = 9.6% (7.2%); and factor 3 (Discrimination) = 6.2% (7.0%).

Some cross-loadings were observed but they were minimal. Most items displayed substantially higher loadings on their primary factor than on others, which supports discriminant validity among the dimensions. Table 3 shows that the majority of items achieved h^2^ > 0.50, and alpha values were high, indicating an adequate proportion of explained variance.

Table 4 summarizes correlations among the FSA subscales. All correlations are statistically significant (Spearman’s ρ, *p* < 0.01), with the strongest associations observed between Avoidance and the total FSA score (r = 0.912), Discrimination and the total FSA score (r = 0.878), and Antilocution and Avoidance (r = 0.729). These findings indicate that the factors are interrelated yet remain conceptually distinguishable.

Composite Reliability (CR) values, reported on Table 4 diagonal, document reliability of the latent constructs. These values exceed the critical threshold of 0.70 [62], further supporting the model’s internal consistency.

### 3.3. Scale Dimensions

**Figure 1:**
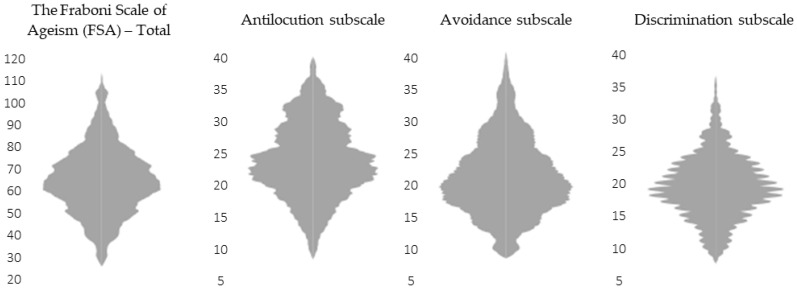
The Fraboni Scale of Ageism (FSA) Distribution. Note: Range of the total FSA Scale is 29–116; range of the antilocution subscale is 13–52; range of avoidance and discrimination subscales are 8–32.

As shown in Figure 1, total FSA score averaged 65.65 (SD = 15.053; median = 65), with near-symmetric distribution (skewness = 0.247) and no pronounced extremes (kurtosis = 0.071). Cronbach’s α = 0.949 indicates excellent internal consistency for the Czech sample. Notably, no floor or ceiling effects (0.0%) were detected, suggesting an even spread of responses across the full-scale range.

The Antilocution subscale yielded M = 24.19 (SD = 5.826; median = 24), with skewness = 0.105 and kurtosis = −0.385, indicating an approximately normal distribution. Internal consistency was high (α = 0.891). Floor effects were minimal (0.6%), and no ceiling effects were observed.

The Avoidance subscale showed M = 21.56 (SD = 5.901; median = 21) with slight positive skew (skewness = 0.471), consistent with a tendency toward lower avoidance among a subset of respondents. Kurtosis was close to zero (−0.034), again indicating an approximately normal distribution. Reliability was very high (α = 0.898). Floor and ceiling effects were 1.8% and 0.2%, respectively.

The Discrimination subscale averaged 19.90 (SD = 4.796; median = 20), with skewness = 0.280 and kurtosis = 0.207, indicating a balanced distribution without extreme clustering. Internal consistency was high (α = 0.848), and no floor or ceiling effects were present.

### 3.4. Psychometric Performance of the FSA

**Figure 2:**
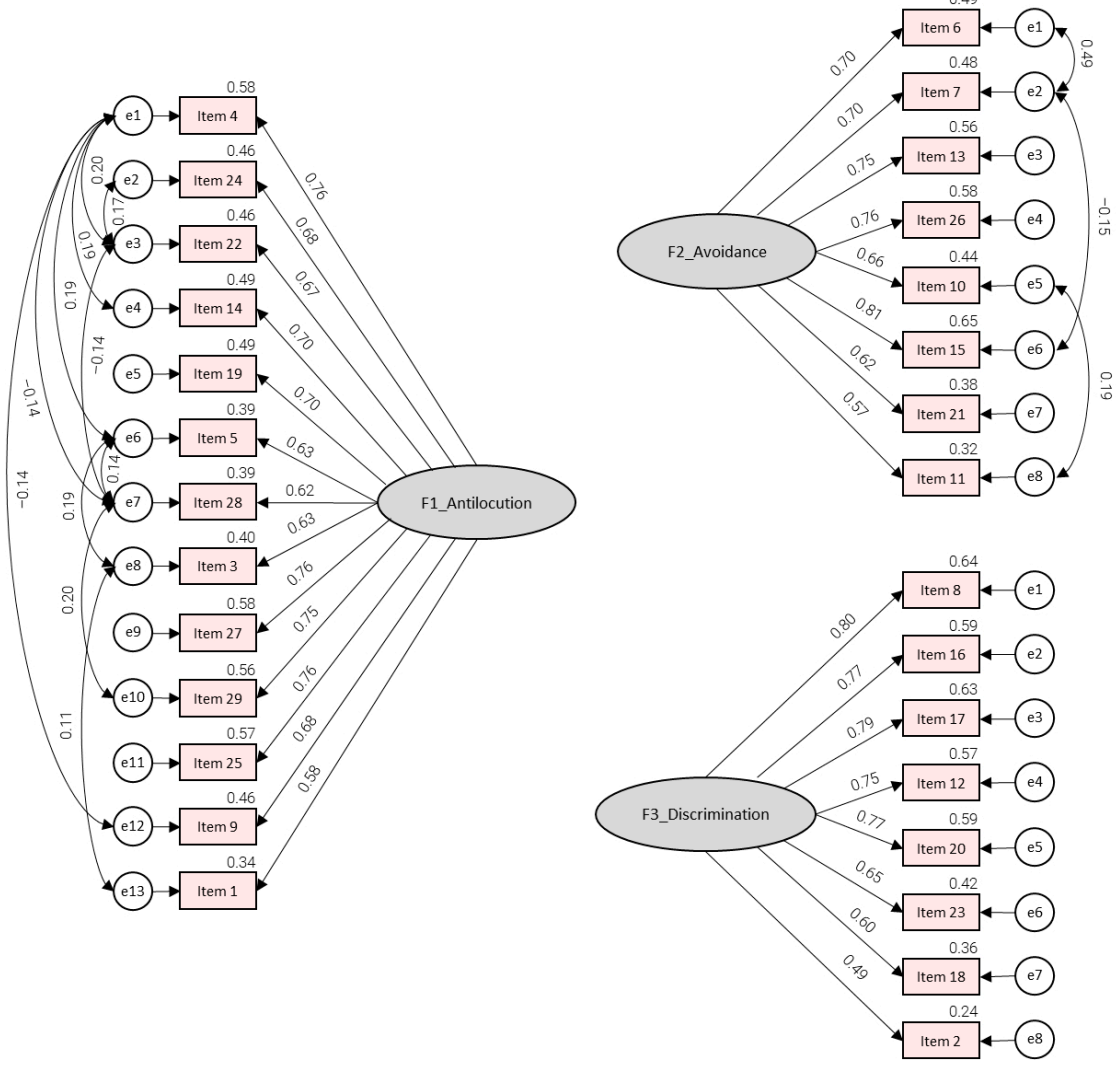
CFA of the improved model.

To verify the factor structure of the Czech version of FSA, confirmatory factor analysis (CFA) was conducted on the second half of the dataset (n = 548). Figure 2 presents the final three-factor CFA model with standardized loadings for the Antilocution, Avoidance, and Discrimination dimensions.

As it is summarized in Table 5, for the Antilocution subscale, the original specification fit the data only adequately: although standardized residuals were acceptable (SRMR = 0.0452), the absolute fit was weak (RMSEA = 0.087; GFI = 0.898), the incremental indices fell below the preferred 0.95 threshold (CFI = 0.925; TLI = 0.910; NFI = 0.909), and the chi-square to degrees-of-freedom ratio was high (χ^2^/df = 331.241/65). After theory-consistent re-specifications guided by modification indices, the improved Antilocution model exhibited good to excellent fit across all criteria: RMSEA dropped to 0.058, SRMR to 0.0326, GFI rose to 0.954, and the incremental indices exceeded 0.95 (CFI = 0.972; TLI = 0.959; NFI = 0.958), with more favorable χ^2^/df.

For the Avoidance subscale, the initial model clearly misfit the data. It was evident in RMSEA = 0.123, the chi-square/df ratio was large (χ^2^/df = 184.920/20), while the incremental indices were marginal (CFI = 0.918; TLI = 0.886; NFI = 0.910), despite an acceptable SRMR = 0.0458 and GFI = 0.921. After targeted modifications reflecting empirically supported residual relations, the model achieved near-ideal fit: the chi-square became non-significant (χ^2^ = 27.502, df = 17, *p* = 0.051), RMSEA fell to 0.034, SRMR to 0.0195, GFI rose to 0.988, and all incremental indices surpassed 0.98 (CFI = 0.995; TLI = 0.991; NFI = 0.987), with low χ^2^/df.

The modification indices were inspected, and a small number of theory-consistent within-factor residual covariances were introduced to account for local item dependencies (see Figure 2). These pairings were deemed substantively defensible because they capture shared semantic or method variance (e.g., clustered inclusion items or related stereotype or discomfort statements) beyond the common latent factor. This improves the fit without altering the intended three-factor structure. No items were removed. Although removing lower-performing items from the descriptive/ITC patterns was considered, reliability diagnostics and the goal of validating the full 29-item instrument supported retaining all items to maintain comparability with the original scale and prior adaptations.

By contrast, the Discrimination subscale demonstrated excellent fit without any modification. The chi-square ratio was low (χ^2^/df = 47.504/20) and both absolute and incremental indices met or exceeded recommended thresholds (RMSEA = 0.050; SRMR = 0.0265; GFI = 0.978; CFI = 0.986; TLI = 0.980; NFI = 0.976).

### 3.5. Convergent Validity

Convergent validity reflects the extent to which items intended to measure the same latent construct are interrelated [63]. A valid scale should therefore comprise items that correlate meaningfully with one another and share common substantive content.

Initial evidence comes from the exploratory factor analysis: items clustered into the three theoretically posited dimensions, with most factor loadings exceeding 0.60 and communalities (h^2^) exceeding 0.50 in most cases. This strong saturation of latent factors, together with the clear item grouping, indicates that items within each dimension tap the same underlying construct. We further assessed convergence using Average Variance Extracted (AVE), which represents the average proportion of variance in the indicators accounted for by the latent factor [55]. The AVE values were 0.48 for antilocution (F1), 0.47 for avoidance (F2) and 0.48 for discrimination (F3).

Although the conventional benchmark is 0.50, values in the 0.45–0.49 range are often deemed acceptable when other validity evidence is strong and Composite Reliability (CR) is high [55]. In our case, all subscales showed very high reliability (CR > 0.88), supporting the acceptability of the slightly sub-threshold AVE results.

Additional support comes from inter-item correlations: within each subscale, item–item associations were statistically significant (*p* < 0.001) and ranged from moderate to high. This pattern demonstrates internal coherence and further corroborates convergence on a common latent factor. Items are meaningfully connected without being redundant.

### 3.6. Construct Validity

The Kendall’s tau-b matrix in Appendix A, Table A1, Table A2 and Table A3, shows positive, and largely moderate web of associations across all FSA items (most τ = 0.20–0.50, all *p* < 0.01), consistent with a common ageism construct while preserving subfacet nuance. The Avoidance cluster is especially tight: items 6–7 (τ = 0.720), 6–13 (0.505), 7–15 (0.508), 13–15 (0.495), and 15–26 (0.564) indicate respondents who admit one distancing behavior tend to admit others. Stereotype-laden Antilocution items also cohere strongly (e.g., 4–5 τ = 0.532; 25–27 τ = 0.565; 27–29 τ = 0.574). Reverse-coded inclusion items behave as expected after re-coding, aligning positively with the stereotype items (e.g., 4–24 τ = 0.670; 4–22 τ = 0.623; 14–19 τ = 0.535), which supports the intended scoring direction. A few items are less central, mirroring earlier ITC findings. Item 16 shows weaker links to several statements (e.g., τ = 0.18–0.36), and item 2 also posts modest correlations (several τ = 0.12–0.26).

### 3.7. Relationship Between the FSA and Respondent Profiles

Further evidence for the applicability of the Czech FSA comes from analyses of relationships between FSA scores and selected respondent characteristics. As summarized in Table 6, the observed patterns align with theory and prior findings, indicating that the FSA sensitively reflects variation in social experience, contact with older adults, fear of old age, and education.

A clear generational gradient emerged, with mean FSA scores increasing systematically from older to younger cohorts: Boomers: 62.73; Generation X: 62.96; Generation Y: 67.36; Generation Z: 73.68. Differences were statistically significant (H = 25.258; *p* < 0.001), indicating higher ageist attitudes among younger generations. This pattern is mirrored on the subscales, with the largest inter-cohort gaps in Antilocution and Avoidance.

Consistent with contact-based explanations, respondents co-residing with someone they perceive as elderly reported lower FSA scores (60.88) than those who do not (66.16). Such contrast was statistically significant (U = 10,629.000; *p* = 0.045).

Convergent validity is strongly supported by the association between fear of old age and FSA scores. FSA values increased with higher fear starting from 44.70 continuing through 60.63 up to the 83.02. The trend was linear with significant differences across levels (H = 187.720; *p* < 0.001).

An inverse relationship with education was observed. Respondents with primary education had the highest FSA scores (71.81), whereas university graduates had the lowest (62.85), a statistically significant difference (H = 10.245; *p* = 0.017).

Finally, respondents who define old age earlier (i.e., 50–65 years) exhibited higher FSA scores (68.44) than those who consider ≥76 or 80 years to mark old age (62.37 and 63.73). The difference was statistically significant (H = 11.643; *p* = 0.009) and most pronounced on Antilocution.

## 4. Discussion

This study set out to evaluate the psychometric properties, reliability, and validity of the FSA in the Czech population, to examine its dimensionality, and to assess whether population attitudes align with the theorized model. Using a multi-method strategy encompassing exploratory factor analysis (EFA), internal-consistency diagnostics, confirmatory factor analysis (CFA), and associations with theoretically relevant variables we achieved these aims. Results indicate that the scale captures its intended dimensions of ageism and that the subscales represent coherent, conceptually consistent constructs. The accumulated evidence supports the convergent validity of the Czech FSA and its use as a valid instrument for measuring ageism in the Czech context.

The Czech FSA demonstrated excellent internal consistency (α = 0.949), with subscale reliabilities ranging from 0.848 to 0.898. Relative to the original scale (Fraboni et al., 1990) [29] which M = 57.89, SD = 11.86, the present study shows higher average ageism scores (M = 65.65), plausibly reflecting culturally specific perceptions of old age in the Czech context and differing intergenerational experiences. Compared to the original study (M = 21.73; SD = 5.77; α = 0.76), both the average score and reliability are higher. In comparison with the original measurement (M = 18.94; SD = 3.08; α = 0.77), the behavioral component appears somewhat more pronounced in the Czech context and exhibits stronger psychometric performance. Relative to the original scale (M = 17.23; SD = 3.42; α = 0.65), declared discriminatory attitudes are higher, and reliability is substantially improved. The three-factor structure identified with PCA was interpretable and congruent with Allport’s (1954) [30] framework of prejudice, capturing Antilocution, Avoidance, and Discrimination. The correlational matrix supports a general ageism factor with three interrelated subdimensions: dense intra-cluster ties for Avoidance, solid links among Antilocution stereotypes, and broadly convergent relations for Discrimination. The pattern aligns with the EFA, and CFA results; the scale is internally coherent and modifications made were justifiable for a few locally dependent or weakly aligned items. CFA corroborated the suitability of this three-factor solution across absolute (RMSEA, SRMR, GFI) and incremental (CFI, TLI, NFI) indices.

Results parallel other cultural adaptations, where strong reliability and a stable three-factor pattern have also been reported [33,34,64,65]. Moreover, convergent evidence in the present study, especially associations with contact, subjective perceptions of old age, fear of old age, and education aligns with prior work showing that the FSA is sensitive to theoretically grounded external correlates. This supports the conclusion that the instrument is psychometrically robust and capable of detecting nuanced expressions of ageist attitudes.

The generational gradient (higher ageism among younger cohorts) accords with social identity theory expectations and echoes patterns observed in earlier studies (e.g., [66,67,68,69,70]). The result indicating lower FSA scores among respondents co-residing with a person they perceive as older is consistent with the contact hypothesis which posits that sustained, meaningful intergroup contact can reduce prejudice, especially on Avoidance dimension [71,72]. Likewise, the association between fear of old age and FSA reinforces the affective basis of prejudice; the strongest associations on Antilocution and Discrimination fit models in which fear operates as a motivational driver of negative stereotyping and support for unequal treatment [73]. An inverse association with education also emerged, which is consistent with the expectation that higher education is related to greater tolerance and openness; in this study it is most visible on Discrimination dimension (Moroianu, 2021) [19]. In terms of sociodemographic differences, women exhibited lower ageism than men, echoing the original validation [29] and other studies [74,75,76].

Although differences by generations are reported, these categories should be interpreted as age-group contrasts in a single cross-sectional snapshot, rather than the evidence of true generational effects. In cross-sectional data, observed differences between Boomers, Gen X, Gen Y, and Gen Z may reflect life-stage processes (e.g., changing contact patterns, roles, and the salience of aging), socialization, or a combination of these factors. These sources of variation cannot be empirically disentangled without longitudinal designs. Accordingly, the observed pattern of higher FSA scores among younger groups indicates where ageism is currently more prevalent rather than implying stable, intrinsic generational dispositions.

Contextualizing the higher Czech mean (and elevated Antilocution scores), cultural conceptions of aging and cohort-specific social experiences likely contribute to stronger endorsement of negative stereotypes. The increased level of self-reported discriminatory attitudes may also reflect institutional settings and historical experiences with ageing and intergenerational solidarity specific to the Czech environment [15,77,78].

Given demographic aging, family atomization, growth of single-person households, and potential intergenerational tensions, routine monitoring with validated tools such as the FSA is warranted. The instrument can inform ageing policy, guide educational programming, and support the evaluation of awareness campaigns aimed at reducing ageism and strengthening intergenerational cohesion [79]. Academically, the study provides evidence for the FSA’s psychometric characteristics in Czechia and confirms its three-factor structure. Practically, it offers a robust measure for ongoing surveillance and intervention assessment [80].

The cross-sectional design precludes causal inference and tracking change over time. Although explained variance was strong, EFA indicates that additional determinants of ageism likely remain. Some items would benefit from further qualitative verification (e.g., cognitive interviewing) to probe interpretation across subgroups. Despite these limitations, results offer strong support for the construct validity of the Czech FSA and indicate its suitability for both research and applied use. Future research might test measurement invariance by gender, age, and education; compare the full FSA with validated short forms; and employ longitudinal or experimental designs to disentangle age, period, and cohort effects and to evaluate intervention impact. Future work can further include the validation of a shortened FSA. Although internal consistency did not indicate the need to remove specific items, exploratory analyses suggest that some statements show relatively high cross-loadings across factors. These items merit targeted revision, clarification, or rewording to enhance conceptual clarity and psychometric performance.

## 5. Conclusions

Developed in Canada in the early 1990s, the Fraboni Scale of Ageism (FSA) remains a widely used instrument for assessing ageist attitudes. The present study demonstrates that the FSA performs well in the Czech context; the scale exhibits very good internal consistency, an adequate factor structure, and strong convergent validity. Taken together, these findings indicate that the Czech version of the FSA is a reliable and valid tool for assessing ageism in the Czech population.

Systematic measurement with instruments such as the FSA enables not only point-in-time monitoring of ageist attitudes, but also the analysis of trends over time and differences between population groups. Such evidence is essential for designing and evaluating policies that promote intergenerational solidarity and reduce age-related prejudice.

In sum, this study confirms the quality of the FSA as a measurement tool and provides a solid foundation for further research on ageism in both the Czech and international contexts.

## Figures and Tables

**Table 1:** Selected socio-demographic characteristics.

Variables		Sample	Population
Gender	Male	50.1%	50.0%
	Female	49.9%	50.0%
	Total	100.0%	100.0%
Generations	Baby Boomers (years)	20.8%	22.7%
	Generation X (years)	29.6%	29.9%
	Generation Y (years)	29.8%	28.3%
	Generation Z (years)	19.8%	19.1%
	Total	100.0%	100.0%
Size of settlement	less than 1000 inhabitants	17.0%	17.3%
	1000 to 4999 inhabitants	22.7%	22.6%
	5000 to 19,999 inhabitants	18.5%	18.1%
	20,000 to 99,999 inhabitants	19.6%	20.2%
	100,000 inhabitants and more	22.2%	21.8%
	Total	100.0%	100.0%

**Table 2:** Descriptive Statistics of the Fraboni Scale of Ageism (FSA) and Its Items.

	Item	n	Mean	SD	Skewness	Kurtosis	ITC
Teenage suicide is more tragic than suicide among the old.	1	544	2.54	1.004	–0.116	–1.055	0.521
There should be special clubs set aside within sports facilities so that old people can compete at their own level.	2	544	2.45	0.776	−0.345	−0.479	0.443
Many old people are stingy and hoard their money and possessions.	3	544	2.39	0.786	0.134	–0.380	0.634
Many old people are not interested in making new friends preferring instead the circle of friends they have had for years.	4	544	2.45	0.837	–0.028	–0.585	0.742
Many old people just live in the past.	5	544	2.53	0.825	–0.118	–0.519	0.651
I sometimes avoid eye contact with old people when I see them.	6	544	1.81	0.837	0.644	–0.543	0.726
I do not like it when old people try to make conversation with me.	7	544	1.90	0.804	0.576	–0.264	0.716
Old people deserve the same rights and freedoms as do other members of our society. *	8	544	1.94	0.879	0.536	−0.612	0.634
Complex and interesting conversation cannot be expected from most old people.	9	544	2.30	0.769	0.120	–0.375	0.666
Feeling depressed when around old people is probably a common feeling.	10	544	2.22	0.815	0.177	–0.540	0.651
Old people should find friends their own age.	11	544	2.38	0.778	0.013	–0.430	0.597
Old people should feel welcome at the social gatherings of young people. *	12	544	2.07	0.822	0.522	–0.132	0.513
I would prefer not to go to an open house at a senior’s club, if invited.	13	544	2.17	0.900	0.380	–0.619	0.704
Old people can be very creative. *	14	544	2.23	0.781	0.171	–0.407	0.569
I personally would not want to spend much time with an old person.	15	544	2.17	0.812	0.245	–0.490	0.725
Most old people should not be allowed to renew their driver’s licenses.	16	544	2.42	0.815	0.063	–0.499	0.398
Old people do not really need to use our community sports facilities.	17	544	2.14	0.813	0.176	−0.662	0.658
Most old people should not be trusted to take care of infants.	18	544	2.42	0.778	−0.012	−0.420	0.549
Many old people are happiest when they are with people their own age.	19	544	2.42	0.744	–0.152	–0.394	0.515
It is best that old people live where they would not bother anyone.	20	544	2.11	0.843	0.426	−0.380	0.653
The company of most old people is quite enjoyable. *	21	544	2.22	0.715	0.316	0.052	0.511
It is sad to hear about the plight of the old in our society these days. *	22	544	2.20	0.794	0.328	−0.253	0.505
Old people should be encouraged to speak out politically. *	23	544	2.17	0.754	0.461	0.136	0.612
Most old people are interesting, individualistic people. *	24	544	2.25	0.774	0.185	−0.350	0.517
Most old people would be considered to have poor personal hygiene.	25	544	2.31	0.771	–0.016	–0.504	0.683
I would prefer not to live with an old person.	26	544	2.19	0.868	0.352	–0.523	0.731
Most old people can be irritating because they tell the same stories over and over again.	27	544	2.34	0.791	0.044	–0.483	0.727
Old people complain more than other people do.	28	544	2.53	0.816	–0.091	–0.495	0.673
Old people do not need much money to meet their needs.	29	544	2.39	0.752	0.080	–0.317	0.673

* Reverse coding. The items are listed here in an organized form, but their order was randomized during the survey. All item statistics are based on listwise deletion of missing cases hence the constant n = 544 across items. Inspection of missing data patterns did not reveal systematic associations with observed variables, so listwise deletion was deemed acceptable.

**Table 3:** Exploratory Factor Analysis (FSA).

Label	Item	n	F1 Antilocution	F2 Avoidance	F3 Discrimination	h^2^
Many old people are not interested in making new friends preferring instead the circle of friends they have had for years.	4	544	0.842			0.757
Most old people are interesting, individualistic people.	24 *	544	0.769			0.667
It is sad to hear about the plight of the old in our society these days.	22 *	544	0.750			0.617
Old people can be very creative.	14 *	544	0.740			0.648
Many old people are happiest when they are with people their own age.	19	544	0.713			0.574
Many old people just live in the past.	5	544	0.707			0.531
Old people complain more than other people do.	28	544	0.698			0.570
Many old people are stingy and hoard their money and possessions.	3	544	0.667			0.500
Most old people can be irritating because they tell the same stories over and over again.	27	544	0.667			0.619
Old people do not need much money to meet their needs.	29	544	0.629			0.541
Most old people would be considered to have poor personal hygiene.	25	544	0.612			0.530
Complex and interesting conversation cannot be expected from most old people.	9	544	0.594			0.488
Teenage suicide is more tragic than suicide among the old.	1	544	0.546			0.337
I sometimes avoid eye contact with old people when I see them.	6	544		0.794		0.706
I do not like it when old people try to make conversation with me.	7	544		0.777		0.679
I would prefer not to go to an open house at a senior’s club, if invited.	13	544		0.725		0.627
I would prefer not to live with an old person.	26	544		0.686		0.649
Feeling depressed when around old people is probably a common feeling.	10	544		0.681		0.568
I personally would not want to spend much time with an old person.	15	544		0.670		0.631
The company of most old people is quite enjoyable.	21 *	544		0.588		0.470
Old people should find friends their own age.	11	544		0.534		0.427
Old people deserve the same rights and freedoms as do other members of our society.	8 *	544			0.777	0.677
Most old people should not be allowed to renew their driver’s licenses.	16	544			0.773	0.649
Old people do not really need to use our community sports facilities.	17	544			0.755	0.658
Old people should feel welcome at the social gatherings of young people.	12 *	544			0.742	0.668
It is best that old people live where they will not bother anyone.	20	544			0.723	0.629
Old people should be encouraged to speak out politically.	23 *	544			0.710	0.569
Most old people should not be trusted to take care of infants.	18	544			0.501	0.394
There should be special clubs set aside within sports facilities so that old people can compete at their own level.	2	544			0.497	0.316
Eigenvalue			12.113	2.783	1.799	

* Reverse coding.

**Table 4:** Correlation among Fraboni Scale of Ageism and Particular Original Subscales.

	Antilocution	Avoidance	Discrimination	FSA
Antilocution	0.92	0.729 **	0.695 **	0.902 **
Avoidance		0.88	0.736 **	0.912 **
Discrimination			0.88	0.878 **

Note: Spearman’s rho; ** *p* < 0.01.

**Table 5:** Absolute and Incremental Indices.

	Chi-Square Test	RMSEA	SRMR	GFI	CFI	TLI	NFI
Antilocution							
Original model	χ^2^ = 331.241, df = 65, *p* < 0.001	0.087	0.0452	0.898	0.925	0.910	0.909
Improved model	χ^2^ = 154.651, df = 54, *p* < 0.001	0.058	0.0326	0.954	0.972	0.959	0.958
Avoidance							
Original model	χ^2^ = 184.920, df = 20, *p* < 0.001	0.123	0.0458	0.921	0.918	0.886	0.910
Improved model	χ^2^ = 27.502, df = 17, *p* = 0.051	0.034	0.0195	0.988	0.995	0.991	0.987
Discrimination							
Original model	χ^2^ = 47.504, df = 20, *p* < 0.001	0.050	0.0265	0.978	0.986	0.980	0.976
Critical values [59]		<0.07	<0.08	>0.95	>0.90	>0.95	>0.95

**Table 6:** Associations of FSA—Total with Auxiliary measures.

	%	Mean	SD	U/H	*p*-Value
Generations				25.258	<0.001
Boomers	20.8%	62.73	14.434		
Generation X	29.6%	62.96	13.325		
Generation Y	29.8%	67.36	15.832		
Generation Z	19.8%	73.68	14.758		
Presence of an older adult in the household				10,629.000	0.045
Yes	9.6%	60.88	13.962		
No	90.4%	66.16	15.089		
Fear of old age				187.720	<0.001
1 (lowest)	1.8%	44.70	10.264		
2	4.6%	47.80	8.093		
3	4.2%	52.39	9.079		
4	8.6%	60.28	13.336		
5	17.3%	60.63	11.667		
6	16.2%	64.44	12.593		
7	15.6%	65.18	10.461		
8	15.6%	72.94	14.223		
9	6.3%	74.29	14.182		
10 (highest)	9.7%	83.02	12.341		
Education				10.245	0.017
Elementary	3.9%	71.81	15.920		
Vocational	32.7%	67.06	14.751		
Secondary	46.1%	65.19	15.516		
University	17.3%	62.85	13.648		
Perceived age of elderly				11.643	0.009
50–65 years	22.8%	68.44	16.252		
66–75 years	46.0%	66.16	14.626		
76–80 years	19.5%	63.73	14.847		
older than 80 years	11.8%	62.37	13.647		

Note: Mann–Whitney U test was used for two-group comparisons; Kruskal–Wallis test for comparisons across more groups.

## Data Availability

The data used to support the findings of this study will be available from the authors upon reasonable request.

## Abbreviations

The following abbreviations are used in this manuscript:

AVE	Average Variance Extracted
CFA	Confirmatory factor analysis
CFI	Comparative Fit Index
CR	Composite Reliability
DF	degrees of freedom
EFA	Exploratory factor analysis
FSA	Fraboni scale of ageism
GFI	goodness of fit index
ITC	item-total correlation
KMO	Kaiser–Meyer–Olkin
NFI	Normed Fit Index
PCA	principal component analysis
RMSEA	Root Mean Square Error of Approximation
SD	standard deviation
SRMR	Standardized Root Mean Squared Residual
TLI	Tucker–Lewis Index

## Appendix A

**Table A1:** Correlational Matrix (Antilocution Items).

	1	3	4	5	9	14	19	22	24	25	27	28	29
1	1.000	0.380	0.393	0.328	0.322	0.362	0.316	0.347	0.371	0.296	0.333	0.358	0.321
2	0.195	0.198	0.237	0.246	0.206	0.183	0.216	0.204	0.228	0.176	0.187	0.146	0.190
3	0.380	1.000	0.497	0.469	0.392	0.420	0.413	0.439	0.467	0.409	0.438	0.380	0.372
4	0.393	0.497	1.000	0.532	0.482	0.643	0.589	0.623	0.670	0.476	0.528	0.533	0.499
5	0.328	0.469	0.532	1.000	0.435	0.434	0.417	0.423	0.434	0.441	0.424	0.412	0.408
6	0.223	0.288	0.316	0.237	0.305	0.378	0.276	0.264	0.346	0.352	0.386	0.286	0.382
7	0.209	0.298	0.306	0.244	0.325	0.362	0.292	0.303	0.373	0.354	0.393	0.303	0.372
8	0.157	0.191	0.194	0.162	0.234	0.266	0.180	0.247	0.250	0.277	0.278	0.175	0.297
9	0.322	0.392	0.482	0.435	1.000	0.435	0.424	0.381	0.405	0.517	0.490	0.412	0.438
10	0.291	0.290	0.313	0.266	0.417	0.312	0.274	0.266	0.296	0.386	0.406	0.315	0.368
11	0.246	0.307	0.335	0.279	0.302	0.339	0.350	0.305	0.323	0.342	0.354	0.344	0.350
12	0.169	0.216	0.269	0.210	0.299	0.291	0.247	0.281	0.280	0.294	0.292	0.220	0.310
13	0.208	0.257	0.278	0.235	0.341	0.356	0.292	0.298	0.342	0.341	0.359	0.323	0.328
14	0.362	0.420	0.643	0.434	0.435	1.000	0.535	0.580	0.623	0.449	0.519	0.476	0.508
15	0.266	0.320	0.388	0.295	0.392	0.399	0.359	0.312	0.408	0.411	0.487	0.412	0.410
16	0.220	0.193	0.217	0.224	0.287	0.257	0.201	0.216	0.209	0.261	0.276	0.257	0.267
17	0.179	0.189	0.232	0.143	0.274	0.284	0.221	0.216	0.248	0.301	0.294	0.244	0.323
18	0.235	0.244	0.303	0.265	0.281	0.311	0.334	0.271	0.275	0.301	0.333	0.292	0.321
19	0.316	0.413	0.589	0.417	0.424	0.535	1.000	0.506	0.521	0.429	0.458	0.511	0.456
20	0.218	0.239	0.305	0.195	0.309	0.314	0.298	0.292	0.279	0.326	0.352	0.280	0.356
21	0.217	0.217	0.267	0.208	0.300	0.360	0.269	0.348	0.337	0.318	0.347	0.278	0.257
22	0.347	0.439	0.623	0.423	0.381	0.580	0.506	1.000	0.616	0.428	0.478	0.458	0.448
23	0.145	0.220	0.265	0.204	0.211	0.297	0.234	0.278	0.275	0.255	0.230	0.202	0.247
24	0.371	0.467	0.670	0.434	0.405	0.623	0.521	0.616	1.000	0.458	0.553	0.493	0.493
25	0.296	0.409	0.476	0.441	0.517	0.449	0.429	0.428	0.458	1.000	0.565	0.454	0.514
26	0.234	0.325	0.377	0.305	0.417	0.424	0.377	0.336	0.389	0.458	0.476	0.373	0.410
27	0.333	0.438	0.528	0.424	0.490	0.519	0.458	0.478	0.553	0.565	1.000	0.517	0.574
28	0.358	0.380	0.533	0.412	0.412	0.476	0.511	0.458	0.493	0.454	0.517	1.000	0.466
29	0.321	0.372	0.499	0.408	0.438	0.508	0.456	0.448	0.493	0.514	0.574	0.466	1.000

**Table A2:** Correlational Matrix (Avoidance Items).

	6	7	10	11	13	15	21	26
1	0.223	0.209	0.291	0.246	0.208	0.266	0.217	0.234
2	0.122	0.160	0.125	0.184	0.165	0.176	0.205	0.190
3	0.288	0.298	0.290	0.307	0.257	0.320	0.217	0.325
4	0.316	0.306	0.313	0.335	0.278	0.388	0.267	0.377
5	0.237	0.244	0.266	0.279	0.235	0.295	0.208	0.305
6	1.000	0.720	0.462	0.378	0.505	0.512	0.436	0.504
7	0.720	1.000	0.435	0.398	0.487	0.508	0.417	0.502
8	0.357	0.359	0.290	0.201	0.316	0.367	0.318	0.347
9	0.305	0.325	0.417	0.302	0.341	0.392	0.300	0.417
10	0.462	0.435	1.000	0.403	0.496	0.478	0.413	0.484
11	0.378	0.398	0.403	1.000	0.413	0.399	0.269	0.374
12	0.402	0.417	0.334	0.279	0.360	0.387	0.332	0.359
13	0.505	0.487	0.496	0.413	1.000	0.495	0.415	0.489
14	0.378	0.362	0.312	0.339	0.356	0.399	0.360	0.424
15	0.512	0.508	0.478	0.399	0.495	1.000	0.431	0.564
16	0.259	0.232	0.248	0.183	0.281	0.314	0.265	0.280
17	0.336	0.318	0.308	0.257	0.333	0.331	0.316	0.331
18	0.236	0.240	0.323	0.266	0.290	0.319	0.293	0.356
19	0.276	0.292	0.274	0.350	0.292	0.359	0.269	0.377
20	0.338	0.324	0.297	0.308	0.331	0.398	0.292	0.362
21	0.436	0.417	0.413	0.269	0.415	0.431	1.000	0.465
22	0.264	0.303	0.266	0.305	0.298	0.312	0.348	0.336
23	0.292	0.284	0.267	0.241	0.293	0.337	0.303	0.270
24	0.346	0.373	0.296	0.323	0.342	0.408	0.337	0.389
25	0.352	0.354	0.386	0.342	0.341	0.411	0.318	0.458
26	0.504	0.502	0.484	0.374	0.489	0.564	0.465	1.000
27	0.386	0.393	0.406	0.354	0.359	0.487	0.347	0.476
28	0.286	0.303	0.315	0.344	0.323	0.412	0.278	0.373
29	0.382	0.372	0.368	0.350	0.328	0.410	0.257	0.410

**Table A3:** Correlational Matrix (Discrimination Items).

	2	8	12	16	17	18	20	23
1	0.195	0.157	0.169	0.220	0.179	0.235	0.218	0.145
2	1.000	0.212	0.260	0.313	0.258	0.294	0.256	0.254
3	0.198	0.191	0.216	0.193	0.189	0.244	0.239	0.220
4	0.237	0.194	0.269	0.217	0.232	0.303	0.305	0.265
5	0.246	0.162	0.210	0.224	0.143	0.265	0.195	0.204
6	0.122	0.357	0.402	0.259	0.336	0.236	0.338	0.292
7	0.160	0.359	0.417	0.232	0.318	0.240	0.324	0.284
8	0.212	1.000	0.597	0.508	0.586	0.320	0.525	0.486
9	0.206	0.234	0.299	0.287	0.274	0.281	0.309	0.211
10	0.125	0.290	0.334	0.248	0.308	0.323	0.297	0.267
11	0.184	0.201	0.279	0.183	0.257	0.266	0.308	0.241
12	0.260	0.597	1.000	0.483	0.531	0.305	0.542	0.515
13	0.165	0.316	0.360	0.281	0.333	0.290	0.331	0.293
14	0.183	0.266	0.291	0.257	0.284	0.311	0.314	0.297
15	0.176	0.367	0.387	0.314	0.331	0.319	0.398	0.337
16	0.313	0.508	0.483	1.000	0.521	0.382	0.469	0.459
17	0.258	0.586	0.531	0.521	1.000	0.391	0.535	0.465
18	0.294	0.320	0.305	0.382	0.391	1.000	0.384	0.305
19	0.216	0.180	0.247	0.201	0.221	0.334	0.298	0.234
20	0.256	0.525	0.542	0.469	0.535	0.384	1.000	0.421
21	0.205	0.318	0.332	0.265	0.316	0.293	0.292	0.303
22	0.204	0.247	0.281	0.216	0.216	0.271	0.292	0.278
23	0.254	0.486	0.515	0.459	0.465	0.305	0.421	1.000
24	0.228	0.250	0.280	0.209	0.248	0.275	0.279	0.275
25	0.176	0.277	0.294	0.261	0.301	0.301	0.326	0.255
26	0.190	0.347	0.359	0.280	0.331	0.356	0.362	0.270
27	0.187	0.278	0.292	0.276	0.294	0.333	0.352	0.230
28	0.146	0.175	0.220	0.257	0.244	0.292	0.280	0.202
29	0.190	0.297	0.310	0.267	0.323	0.321	0.356	0.247
